# Detecting outliers in segmented genomes of flu virus using an alignment-free approach

**DOI:** 10.5808/GI.2020.18.1.e2

**Published:** 2020-03-31

**Authors:** Mosaab Daoud

**Affiliations:** Independent Research Scientist, Toronto, ON M1S1G2, Canada

**Keywords:** composite data point, distance space, flu virus, Mosaab-metric space, outliers, statistical learning

## Abstract

In this paper, we propose a new approach to detecting outliers in a set of segmented genomes of the flu virus, a data set with a heterogeneous set of sequences. The approach has the following computational phases: feature extraction, which is a mapping into feature space, alignment-free distance measure to measure the distance between any two segmented genomes, and a mapping into distance space to analyze a quantum of distance values. The approach is implemented using supervised and unsupervised learning modes. The experiments show robustness in detecting outliers of the segmented genome of the flu virus.

## Introduction

Recent years have witnessed a dramatic increase in the amount of genome data that is submitted to on-line databases. Analyzing sequence-based datasets is the aim of sequence analysis and biodata mining research fields. The engineering solutions have not been achieved to analyze data sets with heterogeneous feature. In other words, the datasets under consideration are sets of sequences with different biological functions and different base-composition distributions. The problem under consideration has several computational challenges. The first challenge is the representation of the inner information structure of a segmented genome of flu virus in feature spaces. Another challenge is to define a metric and metric space to measure the distance between any two information structures that are embedded in a well-defined feature space or composite feature space, and the third challenge is to analyze a quantum of distance values in distance space. The approach that we propose in this paper is alignment-free approach, which is different from classical alignment approaches in terms of time complexity, selectivity, and sensitivity analysis.

At this point, the structure of this paper can be summarized as follows. In next subsection, we shall present a review of the existing approaches to tackle related research problems. In section (Methods), we shall present the approaches of detecting outliers in segmented genomes of the flu virus. The experiments and results are presented in section (Results). Finally, conclusions and future work will be presented in section (Discussion).

### The related work

The Influenza virus is a highly mutated virus. It has a negative impact on the human population. Consequently, it has a negative impact on public health and the economy. The virus has a segmented genome that can be encoded to 10-11 proteins. The virus is classified into types and subtypes. The variation in the base composition of the surface proteins haemagglutinin (HA) and neuraminidase (NA) indicates the type and the subtype of the influenza virus [1].

The influenza virus is a negative stranded RNA-virus. It is classified under the family Orthomyxoviridae. The virus has three types A, B, and C. The most variable type is the influenza virus A compared to other types [2]. The accumulation of point mutation in the HA and NA surface proteins causes an antigenic drift. The evolution process is a continuous or discrete in real time. It takes place on the genetic information of the virus. Consequently, it produces new distinct strains.

An alignment-free sequence comparison analysis is a new developing research direction. It has the potential of solving the sequence proximity problem with less time complexity compared to the alignment-based analysis [3]. There are several strengths behind this fact: we can project those sequences into several feature spaces to detect the information structure in various ways. This approach helps research in bioinformatics and biotechnology fields to gather more information about sequences or genomes. Mapping those sequences into feature spaces in a format of data-vectors allows the computational research community to implement a wide range of techniques in data mining, machine learning, and statistical learning in feature spaces, which are behind the capacity of alignment-based techniques [4]. No prior biological assumptions about sequences are required to implement alignment-free techniques, while the alignment-based techniques have to be implemented with pre-assumptions about the inheritance of sequences. In this context, two concepts are arising: homogeneous and heterogeneous sequences under consideration. Moreover, the alignment-free techniques can be implemented when the alignment-based techniques are inapplicable.

Any biosequence is linear in time. Therefore, the sequential relation is the most promising feature in biosequences [5]. Biosequences are drawn from finite alphabets. Any biosequence can be mapped into a feature space using *n*-grams technique as feature extraction technique. The computational mechanism of this technique can be implemented in different ways. Without loss of generality, assume that we have a sliding window of length *W*, moving a sliding window from one end to another to estimate the relative frequency of the occurrences of *n*-grams. The sliding window can be shifted by a shift distance α. Local statistical information about biosequences can be extracted in this way. The distance between any two sequences can be measured in a feature space by measuring the distance between the frequency distributions (i.e., data vectors) of the two sequences. There are several similarity/distance measures that can be used to measure the distance between data-vectors that are extracted using *n*-grams. The extraction can be achieved either using frequency distribution or relative frequency distribution. A distance function D() is a mapping from a well defined domain to measure the proximity between two entities (e.g., two vectors or sequences) into the interval [0,∞]. D() is a metric if it is satisfying the following conditions: positivity, symmetry, and triangular inequality [5]. The similarity measure S() is a mapping into the interval [0, 1], where the value 0 represents the lowest similarity and the value 1 represents the highest similarity. There are a number of distance measures that can be implemented in measuring the proximity between any two sequences without using alignment. Those measures are either similarity measures or distance measures.

One of the distance measures used in multivariate analysis is the angle cosine between two data-vectors [6]. Each data-vector represents a sequence, and the proximity of two sequences is measured by the angle cosine. The measure detects the differences between two data-vectors, where each data vector represents the relative occurrences of selected *n*-grams. The measure is not sensitive to repetition of motifs. In information theory, the Kullback-Leibler discrepancy is a well-known measure and it measures the divergence between two probability distributions, where each probability distribution represents a sequence, and defined as the occurrences of selected *n*-grams in a sequence.

Han et al. [7] proposed an alignment-free sequence comparison method to detect the dissimilarity between any two sequences. The defined distance is based on two factors: the relative frequency distribution of *n*-grams as a data-vector and the position information as a normalized average data-vector. The distance measure is defined as a weighted distance measure, and the weights are defined in terms of variations of those two factors in a selected genome set. The computational mechanism used in this method is a window-based mechanism. Finally, the phylogenetic tree is composed based on the distance values of the proposed distance measure.

Daoud [8] proposed an alignment-free sequence comparison technique to analyze sequences in feature space. The stochastic membership values of a query sequence with respect to different classes of sequences are estimated using Minkowski measure. The working mechanism proposed in this research is window-based mechanism. The membership value is estimated based on the following question: Is a query sequence probably approximately belongs to a specific class of sequences? In this case, the quantum of distance values composes an empirical distance distribution and the membership value is estimated from the empirical distance distribution.

Daoud [9] proposed a visualization approach to visualize composite data points in feature spaces using the variation theory. The implementation of this computational approach is directed to segmented genomes. It is based on window-based mechanism. The robustness of this approach is implicitly depending on its implementation to flu virus. It is the first attempt to graphically show the serious of difference among the segmented genome of the flu virus.

The other measure used in measuring the distance between two sequences without using alignment is the Euclidean distance [5]. The first step is to extract frequency data-vectors for each sequence using *n*-grams feature extraction technique, then the Euclidean distance is applied to measure the distance between two frequency data-vectors, which reflects the identicalness between two sequences.

As we mentioned in this paper, alignment-free is new developing research direction. There are more than 45 alignment-free tools available with different applications in the area of sequence-analysis. A summary of comparisons between alignment-free and alignment-based algorithms are given in Table 1. As an expectation, the next generation of computational pipelines will use those computational algorithms and tools to achieve fast and reliable computations in sequence analysis.

In this paper, we are focusing on detecting outliers in composite data points (e.g., segmented genome of flu virus). An outlier is a data point that diverge from the majority of other data points in terms of its measured features [12]. In addition, finding patterns of data points that do not confirm to the expected feature measurements are a research challenge. There are many applications to the outlier’s detection, for example, detecting tumors in magnetic resonance imaging, finding frauds in health care insurance, or detecting biodiversity in viruses.

In this section, we presented the most popular alignment-free techniques, and a brief introduction about outlier detection. In the next section, we shall present an outlier detection approach for identifying anomalies in segmented genomes of the flue virus.

## Methods

In this section, we shall present an approach to identify outliers in a dataset of composite data points. A composite data point is a dataset (e.g., set of data-vectors or set of sequences). We shall present two directions: (1) supervised and (2) unsupervised learning modes. In case of the existing training data, the approach can be designed using sequential computational phases. The first computational phase is to map each composite data point into a feature space by defining (p×1) feature vector. Each composite data point can be mapped into a set of data vectors. Those data vectors are extracted from heterogeneous sequences; therefore, the base composition of nucleotide distribution is expected to be heterogeneous. In this context, the next phase is to build an information structure for each composite data point. One of the most popular information structures is the variance-covariance structure. Measuring the distance between any two information structures can be achieved by defining a distance measure or metric. The metric space is defined as a metric and a class of matrices, where each matrix represents an information structure of a set of data vectors with unknown distribution.

Daoud [13] proposed a solution for the composite data points proximity problem. The solution defined a new metric space (Ψ,D_ij_ (γ_1_)), where Ψ is a class of composite data points, and D_ij_ (γ_1_) is a metric. D_ij_ (γ_1_) is defined as follows:

(1) $$D_{ij}(\gamma_{1})=\left|\gamma_{1}^{'}\left(\sum_{X_{n}}^{\left(i\right)}-\sum_{X_{n}}^{\left(j\right)}\right)\gamma_{1}\right|=\left|\lambda_{1}\right|>0$$

where λ_1_ is the largest generalized eigenvalue (associated with the generalized eigenvector γ_1_) of the matrix $\left(\sum_{X_{n}}^{\left(i\right)}-\sum_{X_{n}}^{\left(j\right)}\right)$, where X_n_ is random vector that measures the occurrences of *n*-grams in two composite data points i and j, such that each one represents an instance of a segmented genome of a flu virus.

Measuring the distance between any two variance-covariance matrices Σ^(i)^ and Σ^(j)^ of the same random vector will results distance values. Those distance values represent a random variable. In case of considering more than one feature mapping or feature vector (i.e., projected data into more than one feature space), in this case those distance values represent a random vector. The random vector is a random distance vector and it has a distribution with statistical characteristics, and in this context, we define the concept of the distance distribution paradigm (for more details, see Daoud and Kremer [14]).

**Fig. 1.**
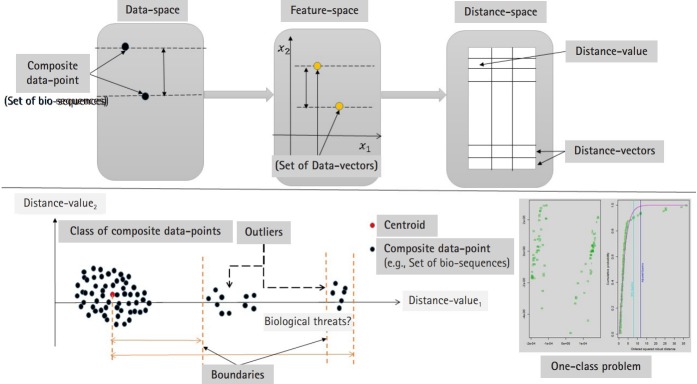
The sketch of the proposed computational model. The upper part represents the abstract of mapping, while the lower part represents the problem under analysis and the expected output.

**Fig. 2.**
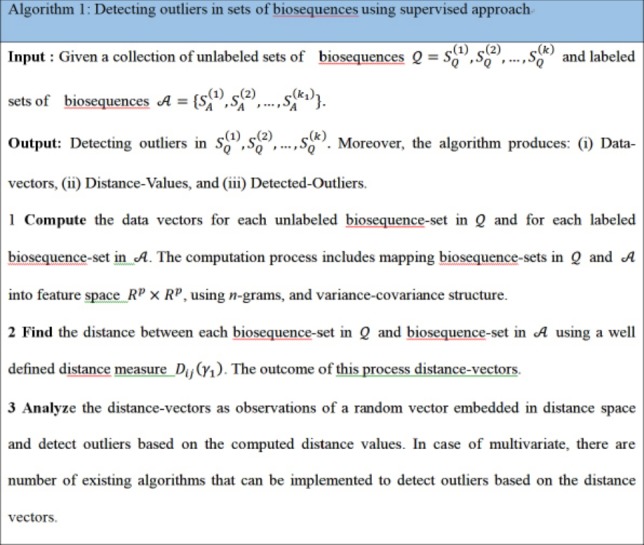
Algorithm 1: Detecting outliers in sets of biosequences using supervised approach.

**Fig. 3.**
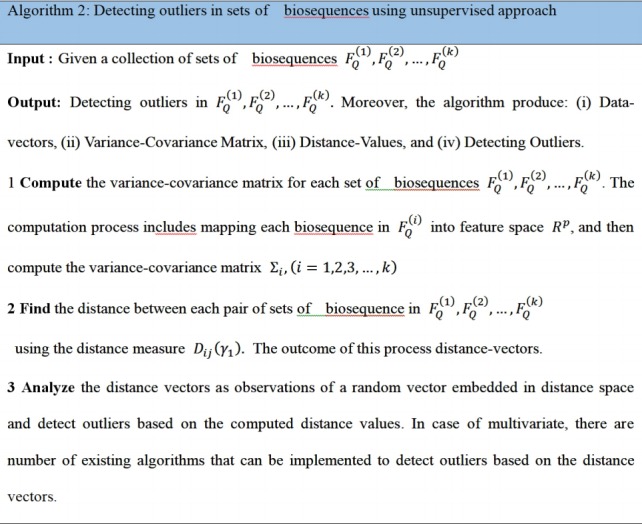
Algorithm 2: Detecting outliers in sets of biosequences using unsupervised approach.

Now consider the analysis of distance values as another phase to integrate the computational process to detect the outliers in distance space. In case of multivariate distance-random vector, there are different multivariate techniques that can be implemented in detecting outliers in distance space. By considering those distance-data vectors as observations of a random vector, we can implement one of the multivariate outlier-detection techniques, which is based on Mahalanobis distance. In this context, the outlier can be defined as a data-vector with largest squared Mahalanobis distance. The empirical distribution function of the ordered square distances and theoretical distribution function, in this case χ^2^-distribution, can be compared to identify outliers with a specific threshold value (quantile) [15,16]. The computational phases are illustrated in Fig. 1, which are in certain way identical to deep learning approach in the sense of using composite feature spaces [17]. Finally, the sketch of the proposed computational techniques (supervised and unsupervised modes) are shown in Figs. 2 and 3 , respectively.

### The validity of the proposed computational approaches

The proposed computational approaches are bio-data mining approaches and it is build upon using data-vectors extracted from biosequences based on *n*-grams features, those features are numerical features. The numerical features represent the biological features. The main contribution of this paper is to propose a new computational framework to detect outliers in composite data points using distance space. The extracted distance vectors in the composite feature space are multivariate random vectors. Then implementing the existing multivariate outliers’ techniques on those distance data-vectors is a validated computational process. In fact, there is no-need to validate those existing multivariate statistical outlier detection techniques.

## Results

In this section, we shall present the robustness of the proposed approaches by showing two experiments. In the fist experiment, we downloaded 47 and 46 segmented genomes of the flu virus A and B respectively, from NCBI website [18]. Those segmented genomes are collected between May and December 2016. The first computational phase, the segmented genomes mapped into three feature spaces. Those feature spaces are (1) 1-grams (bases), (2) 2-grams (dimers), and (3) 3-grams (codons). The dimensionality of the considered feature spaces is: 4, 16, and 64. As a second computational phase, the sets of extracted data-vectors are mapped into the form of variance-covariance matrices. By implementing the metric given in Eq. (1). we measured the proximity of any two variance-covariance matrices using the largest generalized eigenvalue. In the third phase, we analyzed the distance values as observations of (3 ×1) random vector. Based on the squared Mahalanobis distance of the distance data-vectors, the segmented genomes with largest squared Mahalanobis distance are given in Table 2. In the second experiment, we implemented the unsupervised approach to detect outliers in the same dataset of segmented genomes, and the segmented genomes with largest squared Mahalanobis distance are shown in Table 2.

**Fig. 4.**
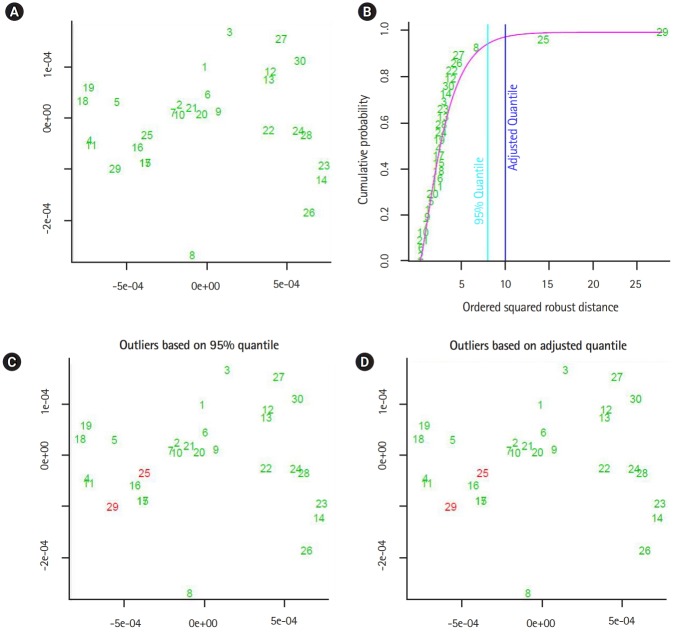
The output from implementing the proposed supervised outlier detection approach (flu virus A). (A, B) The sub-graphs represent the scatter diagram and the distance distribution of composite data points respectively. (C, D) The sub-diagrams represent outlier detections using different quintiles. The figures are generated by using R-package: mvoutlier. We use the function aq.plot to process the distance data-vectors. In addition, left-upper subfigures showing the data projected into two-dimensional space using the first and second principal components.

**Fig. 5.**
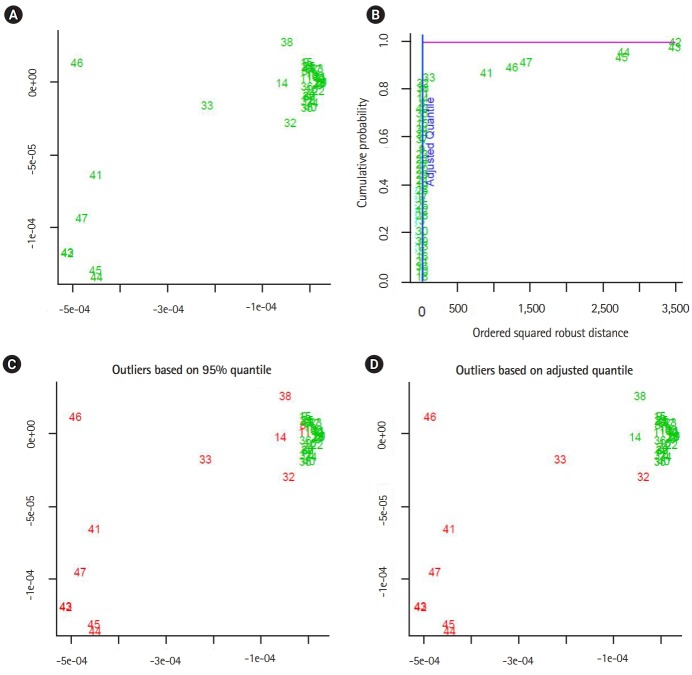
The output from implementing the proposed unsupervised outlier detection approach (flu virus A). (A, B) The sub-graphs represent the scatter diagram and the distance distribution of composite data points respectively. (C, D) The sub-diagrams represent outlier detections using different quintiles. The figures are generated by using R-package: mvoutlier. We use the function aq.plot to process the distance data-vectors. In addition, panel A showing the data projected into two-dimensional space using the first and second principal components.

**Fig. 6.**
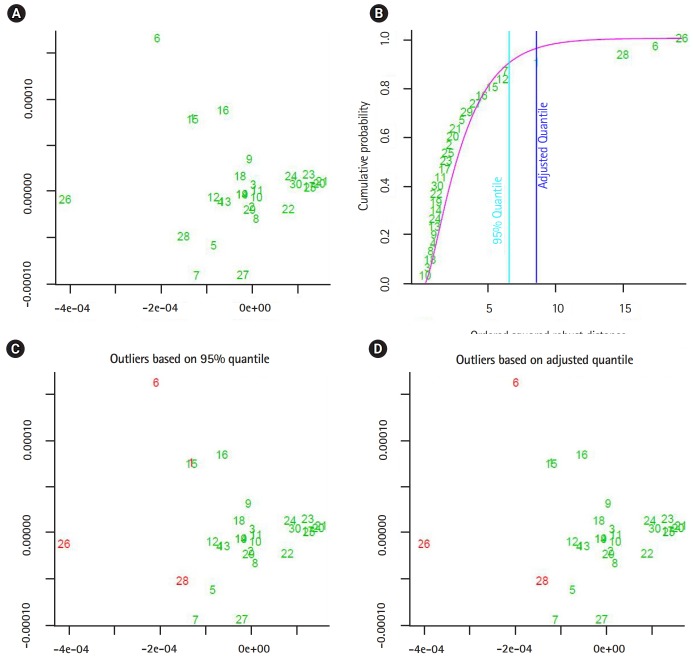
The output from implementing the proposed supervised outlier detection approach (flu virus B). (A, B) The sub-graphs represent the scatter diagram and the distance distribution of composite data points respectively. (C, D) The sub-diagrams represent outlier detections using different quintiles. The figures are generated by using R-package: mvoutlier. We use the function aq.plot to process the distance data-vectors. In addition, left-upper subfigures showing the data projected into two-dimensional space using the first and second principal components.

**Fig. 7.**
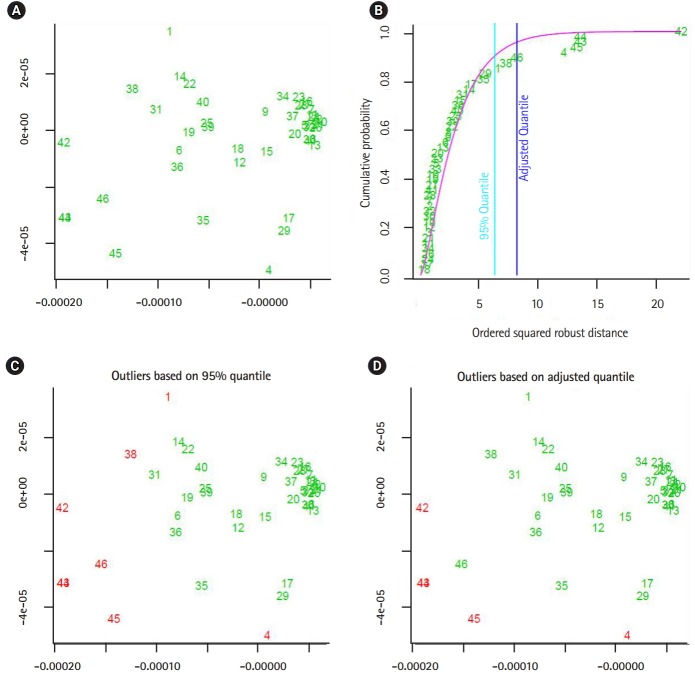
The output from implementing the proposed unsupervised outlier detection approach (flu virus B). (A, B) The sub-graphs represent the scatter diagram and the distance distribution of composite data points respectively. (C, D) The sub-diagrams represent outlier detections using different quintiles. The figures are generated by using R-package: mvoutlier. We use the function aq.plot to process the distance data-vectors. In addition, panel A showing the data projected into two-dimensional space using the first and second principal components.

The output of the proposed approach is illustrated in Figs. 4–7. The outliers of segmented genomes are identified efficiently using supervised learning approach compared with unsupervised learning approach (Table 2). This conclusion is inferred based on the number of identified outliers.

Finally, in this section, we presented the results of implementing the proposed outlier detection approach. In next section, we shall present conclusions and future work.

## Discussion

In this paper, we proposed a new approach to detect outliers in segmented genomes of the flu virus. The flu virus has eight segments that can be encoded into 10–11 proteins, where each protein has different biological function and consequently has different nucleotide composition. Those segmented genomes are heterogeneous by nature. The computational challenges are solved in systematic approach, as feature mapping into the feature space, composite feature representation as variance-covariance matrices, defining a metric space to measure the distance between any two variance-covariance matrices, and finally analyzing those distance-values in the feature space. To evaluate the approach, we implemented it using two datasets: (1) 47 segmented genome of the flu virus A and (2) 46 segmented genomes of the flu virus B. The output of the proposed approach shows the difference between supervised learning and unsupervised learning, and we identified the weaknesses and strengths of each learning mode.

## Figures and Tables

**Table 1.** Comparison between alignment-free and alignment-based techniques [3,5,10,11]

Feature	Alignment-based	Alignment-free
Input data	Sequences	Data-vectors
Assumptions about data	Required	Not-required
Computational scheme	Dynamic programming	Distance-measures
Time complexity	Quadratic	Linear
Applications	Sequence comparison	Sequence comparison
	Phylogenetic tree	Phylogenetic tree
	Function prediction	General mapper
	Genome assembly	Genome assembly
	Reads correcting errors	Reads error correction
	-	Transcript quantification
	Metagenomics	Metagenomics

**Table 2.** The output of supervised and unsupervised learning in detecting the outliers of segmented genomes

Learning approach	Outliers of outliers of flu virus A	Outliers of outliers of flu virus B
Supervised	25, 29	28, 6, 26
Unsupervised	33, 41, 46, 47	46, 4, 45
	45, 44, 43, 42	43, 44, 42
